# Haemoglobin concentration and volume of intravenous fluids in septic shock in the ARISE trial

**DOI:** 10.1186/s13054-018-2029-6

**Published:** 2018-05-03

**Authors:** Matthew J. Maiden, Mark E. Finnis, Sandra Peake, Simon McRae, Anthony Delaney, Michael Bailey, Rinaldo Bellomo

**Affiliations:** 1 Intensive Care Unit, University Hospital Geelong, Barwon Health, PO Pox 281, Geelong, Victoria Australia; 2 Intensive Care Unit, Royal Adelaide Hospital, Adelaide, South Australia Australia; 3 Discipline of Acute Care Medicine, University of Adelaide, Adelaide, Australia; 4 Department of Intensive Care Medicine, The Queen Elizabeth Hospital, Adelaide, South Australia Australia; 5 Department of Haematology, SA Pathology, Adelaide, South Australia Australia; 6 Intensive Care Unit, Royal North Shore Hospital, St Leonard’s, New South Wales Australia; 7 Australian and New Zealand Intensive Care Research Centre, Monash University, Clayton, Victoria Australia; 8 Northern Clinical School, Sydney Medical School, University of Sydney, Clayton, Australia; 9 Critical Care Services, Monash Health, Clayton, Victoria Australia; 10 School of Medicine, University of Melbourne, Melbourne, Victoria Australia; 11 Department of Intensive Care, Austin Hospital, Melbourne, Australia; 12 Department of Intensive Care, Royal Melbourne Hospital, Melbourne, Australia

**Keywords:** Septic shock, Haemoglobin, Fluids, Haemodilution, Resuscitation

## Abstract

**Background:**

Intravenous fluids may contribute to lower haemoglobin levels in patients with septic shock. We sought to determine the relationship between the changes in haemoglobin concentration and the volume of intravenous fluids administered during resuscitation from septic shock.

**Methods:**

We performed a retrospective cohort study of patients enrolled in the Australasian Resuscitation in Sepsis Evaluation (ARISE) trial who were not transfused red blood cells (N = 1275). We determined the relationship between haemoglobin concentration, its change over time and volume of intravenous fluids administered over 6, 24 and 72 h using univariate and multivariate analysis.

**Results:**

Median (IQR) haemoglobin concentration at baseline was 133 (118–146) g/L and decreased to 115 (102–127) g/L within the first 6 h of resuscitation (*P* < 0.001), 110 (99–122) g/L after 24 h, and 109 (97–121) g/L after 72 h. At the corresponding time points, the cumulative volume of intravenous fluid administered was 1.3 (0.7–2.2) L, 2.9 (1.8–4.3) L and 4.6 (2.7–7.1) L. Haemoglobin concentration and its change from baseline had an independent but weak association with intravenous fluid volume at each time point (*R*^2^ < 20%, *P* < 0.001). After adjusting for covariates, each litre of intravenous fluid administered was associated with a change in haemoglobin concentration of − 1.0 g/L (95% CI −1.5 to −0.6, *P* < 0.001) at 24 h and − 1.3 g/L (− 1.6 to − 0.9, *P* < 0.001) at 72 h.

**Conclusions:**

Haemoglobin concentration decreases during resuscitation from septic shock, and has a significant but weak association with the volume of intravenous fluids administered.

**Electronic supplementary material:**

The online version of this article (10.1186/s13054-018-2029-6) contains supplementary material, which is available to authorized users.

## Background

Intravenous fluid administration is a cornerstone in the resuscitation from septic shock [1]. Circulating blood volume may be deficient in septic shock due to plasma extravasation through compromised endothelium [2, 3], alteration of vascular muscle tone leading to redistribution of blood and expanded venous capacitance [4, 5] and other sources of fluid loss such as vomiting, diarrhoea, sweating and insensible losses, thus justifying such intravenous fluid therapy.

Haemoglobin is a large intravascular molecule that, in the absence of bleeding, typically remains within the circulation. An increase of haemoglobin concentration in septic shock, without a red cell transfusion, may identify a relative deficit of circulating plasma volume. Conversely, a decrease in haemoglobin concentration may represent an accumulation of intravascular plasma volume following intravenous fluid administration [6].

An increased haemoglobin concentration is one of the most consistent changes seen following induction of sepsis in many experimental animal models [7–12], and some researchers have titrated intravenous fluids according to this haemoconcentration [7, 13]. In the clinical setting, red cell concentration has been recommended as a guide for fluid replacement in systemic inflammatory diseases such as pancreatitis [14–16] and burn injury [17]. However, few clinical studies have specifically assessed changes in haemoglobin concentration during resuscitation from septic shock [18, 19], and the extent to which this may be related to intravenous fluid volume remains uncertain.

The Australasian Resuscitation in Sepsis Evaluation (ARISE) trial evaluated “early goal-directed therapy” (EDGT) against “usual care” in patients with early septic shock [20, 21]. From the patients enrolled in ARISE, we sought to describe the relationship between haemoglobin concentration and its change over time with the volume of intravenous fluids. In particular, we hypothesised that the volume of administered fluid would have significant correlation with a decline in haemoglobin concentration. A secondary objective was to determine the association between baseline haemoglobin, early changes in its concentration and patient outcomes.

## Methods

### Study design

We conducted a retrospective analysis of a patient cohort from the ARISE trial. Full details of the ARISE trial have been published elsewhere [20, 21]. In brief, the ARISE trial was an Australian and New Zealand Intensive Care Society (ANZICS) Clinical Trials Group and Australasian College for Emergency Medicine endorsed, international multi-centre randomised controlled study, which enrolled 1600 patients presenting to the Emergency Department with early septic shock between 2008 and 2014. Patients with suspected or confirmed infection and two or more criteria for systemic inflammatory response were enrolled if they had (i) hypotension (systolic blood pressure less than 90 mmHg or mean arterial pressure less than 65 mmHg, despite at least 1000 mL of intravenous fluids administered within 60 min) or (ii) blood lactate of 4 mmol/L or greater. Patients were randomly allocated to receive EGDT or usual care and had physiological and treatment parameters (including haemoglobin concentration and intravenous fluids) monitored for 72 h. The primary outcome was mortality at 90 days.

### Study cohort

From the ARISE dataset, we excluded patients who refused to consent to participate, received a red blood cell transfusion during the 72 h study period, had primary polycythaemia, or had a concurrent diagnosis of acute bleeding. Patients with a co-diagnosis of pulmonary oedema at enrolment were also excluded, given this is known to alter circulating red blood cell concentration [22].

### Variables and outcomes

Haemoglobin concentration was measured at study baseline (0 h), hourly as clinically indicated during the next 6 h of resuscitation, and again after 24 and 72 h. The volume of intravenous fluids administered over each time interval (0 to 6 h, 0 to 24 h, 0 to 72 h) was recorded. Intravenous fluids included crystalloids (0.9% saline, 5% dextrose, 4% dextrose + 0.18% saline, Hartmann’s solution), colloids (albumin, Gelofusine^**®**^, starch, other) and blood products not containing red blood cells.

Patient outcomes included duration of invasive mechanical ventilation, length of stay in the ICU and hospital and mortality after 28 and 90 days.

### Analysis

Data are presented as number (percentage), mean (standard deviation, SD) for normally distributed data, or median (interquartile range, IQR) otherwise, with group comparisons by the chi-squared test for equal proportion, Student *t* test or Wilcoxon rank-sum test, respectively. Mean differences are presented with 95% confidence intervals (95% CI) and *P* value. Ventilation duration and ICU and hospital length of stay were markedly skewed, so these variables were log-transformed prior to analysis and are reported as the percentage change (95% CI) derived from the ratio of geometric means. Univariate and multivariate relationships were assessed by linear and logistic regression for continuous and binary outcomes; longitudinal data with repeated measures were assessed using general estimating equations. Model effects are presented as the point estimate (95% CI and *P* value); *R*-squared values are included for ordinary least squares regression. There was no imputation for missing data.

Multivariable models were adjusted for the a priori defined baseline confounders Acute Physiology and Chronic Health Evaluation II (APACHE-II) score, age, gender, weight, Charlson Comorbidity Score (0, 1–2, ≥3), volume of intravenous fluid administered prior to enrolment, study group (EGDT or usual care), systolic blood pressure, serum lactate and creatinine, use of a vasoactive agent at baseline (noradrenaline, adrenaline, metaraminol, dopamine, dobutamine, vasopressin, other) and site of infection (blood, lung, abdomen, urinary, central nervous system, soft tissue, other, unknown). Variables with a *P* value <0.05 were included in a multivariable model analysis.

Sensitivity analyses incorporated the proportion of fluids as crystalloid, central venous pressure (dichotomised as < 10 vs. ≥ 10 mmHg), lactate (< 2 vs. ≥ 2 mmol/L), fluid balance after 72 h (total intravenous fluids administered less the total volume of urine collected), serum creatinine and bilirubin (at 72 h).

Exploratory analyses were conducted to determine if the change in haemoglobin concentration at each hour during the initial 6 h of resuscitation was associated with the volume of intravenous fluids, the type of fluid (colloid or crystalloid), insertion of central venous or arterial cannulae, mechanical ventilation (invasive and non-invasive) or the use of a vasoactive infusion. Analysis was performed with Stata MP/14.2 and Prism 7 software, and a two-sided *P* value of 0.05 was used to indicate statistical significance.

## Results

### Cohort description

Of the 1600 patients enrolled in ARISE, those who refused to consent to participate (N = 9), had received transfused red cells (N = 281), had polycythaemia (*N* = 4), had active gastrointestinal bleeding (N = 1) or pulmonary oedema (N = 30) were excluded. This left a cohort of 1275 patients whose demographics, clinical characteristics, treatments provided and outcomes are summarised in Tables 1 and 2.

**Table 1:** Cohort characteristics at enrolment

Characteristic	Cohort (*N* = 1275)
Age – years (IQR)	65 (51–75)
Male – *N* (%)	782 (61.3%)
Weight – kg (IQR)	77 (65–90)
Charlson Comorbidity Index – score (IQR)	1 (0–2)
APACHE-II^a^ – score (IQR)	14 (10–19)
Receiving mechanical ventilation – *N* (%)	
Invasive	105 (8.2%)
Non-invasive	84 (6.6%)
Systolic pressure – mmHg (IQR)	95 (85–110)
Receiving a vasoactive agent infusion^b^ – *N* (%)	187 (14.7%)
Intravenous fluids administered prior to enrolment^c^	
Volume – L (IQR)	2.5 (1.7–3.3)
Volume per weight – mL/kg (IQR)	31.8 (19.4–45.2)
Serum lactate – mmol/L (IQR)	3.9 (2.1–5.2)
Serum creatinine – μmol/L (IQR)	128 (93–195)
Randomisation group^d^ – *N* (%)	
Early goal directed therapy	614 (48.2%)
Usual care	661 (51.8%)
Source of sepsis – *N* (%)	
Blood	110 (8.6%)
Lung	452 (35.5%)
Abdomen	98 (7.7%)
Urinary	261 (20.5%)
Central nervous system	16 (1.3%)
Soft tissue	127 (10.0%)
Other	98 (7.7%)
Unknown	113 (8.9%)

^a^Acute Physiology and Chronic Health Evaluation-II (APACHE-II) score was calculated from data at randomisation into the ARISE study group

^b^Infusion of vasoactive agents included noradrenaline, adrenaline, metaraminol, phenylephrine and/or dopamine for at least 30 min prior to enrolment

^c^Total intravenous fluid volume prior to enrolment included those given by ambulance personnel and in hospital

^d^There were 796 patients randomised to early goal-directed therapy (EGDT) and 804 to usual care in the ARISE study. Exclusion criteria for this cohort study applied to 182 from the EGDT study group and 143 from the usual care group

**Table 2:** Treatments provided during the 72 h following enrolment and outcomes of the study cohort

Treatments and outcomes	Cohort (*N* = 1275)
Treatments	
Central venous cannula inserted – *N* (%)	1053 (82.6%)
Arterial cannula inserted – *N* (%)	1082 (84.9%)
Admitted to ICU – *N* (%)	1092 (85.6%)
Received surgery – *N* (%)	117 (9.2%)
Received mechanical ventilation^a^ – *N* (%)	480 (37.6%)
Received an infusion of a vasoactive agent^b^ – *N* (%)	858 (67.3%)
Duration of vasoactive agent infusion – hours (IQR)	29 (12–57)
Outcomes	
Invasive ventilation	
Number that received invasive ventilation^c^ – *N* (%)	352 (27.6%)
Duration of invasive ventilation – hours (IQR)	61 (22–163)
Length of stay – days (IQR)	
ICU	2.6 (1.3–5.0)
Hospital	8.1 (4.9–15.3)
Mortality – *N* (%)	
ICU	109 (8.5%)
Hospital	162 (12.7%)
Day 28	163 (12.8%)
Day 90	193 (15.1%)

^a^Mechanical ventilation included invasive and non-invasive modes

^b^Infusion of vasoactive drugs included noradrenaline, adrenaline, metaraminol, phenylephrine and/or dopamine for at least 30 min

^c^Invasive mechanical ventilation at any time during hospital admission

**Fig. 1:**
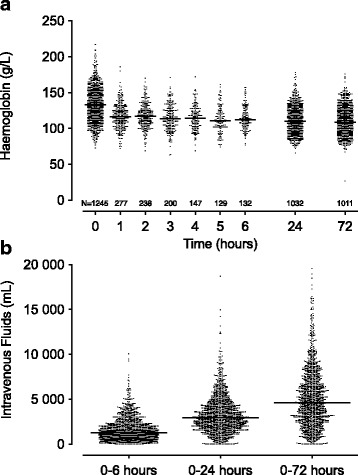
**a** Haemoglobin concentration in a cohort of 1275 patients with septic shock enrolled in the ARISE study (who were not transfused red blood cells). Haemoglobin was measured at baseline (0 h), as required over the next 6 h and then again at 24 h and 72 h. Haemoglobin concentration decreased over time (*P* < 0.0001). **b** Cumulative volume of intravenous fluids administered. Fluid administered before enrolment into the ARISE study were not incorporated. Solid lines represent the median. N, number of patients with haemoglobin concentration recorded

The haemodynamic profile of the cohort during the 72-h period is outlined in Additional file 1: Figure S1. At baseline, the median haemoglobin concentration was 133 (118–146) g/L (137 (121–150) g/L in male participants versus 127 (115–139) g/L in female participants (*P* < 0.001)). Haemoglobin concentration was measured in 808 (63.3%) patients during the first 6 h of resuscitation, within which time it decreased to 115 (103–127) g/L (*P* < 0.001). After 24 h and 72 h, the median haemoglobin concentration was 110 (99–122) and 109 (97–121), respectively (Fig. 1a). Haemoglobin concentration did not differ between ARISE study groups at any time point in this cohort (Additional file 2: Figure S2). The median cumulative volume of intravenous fluids administered was 1.3 (0.7–2.2) L during the first 6 h of resuscitation, 2.9 (1.8–4.3) L after 24 h and 4.6 (2.7–7.1) L after 72 h (Fig. 1b).

### Association between haemoglobin and intravenous fluid volume

Patients with a higher haemoglobin concentration at baseline received slightly more intravenous fluids. For each extra gram of haemoglobin (per litre) at baseline, 6 mL more intravenous fluid was administered over 6 h, 13 mL over 24 h and 22 mL over 72 h. These associations were significant but very weak (*R*^2^ < 5%, *P* < 0.001) in univariate and multivariate analysis at each time point (Additional file 3: Table S1).

**Fig. 2:**
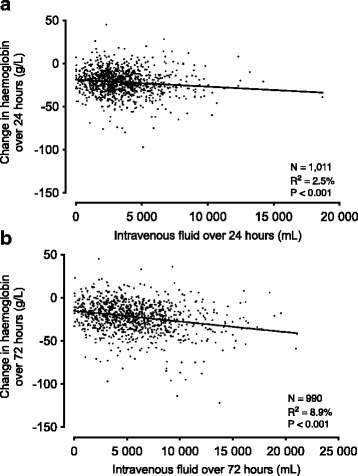
Regression line shows the relationship between the volume of intravenous fluids administered and the corresponding change in haemoglobin concentration during 24 h (**a**), and 72 h (**b**). N, number of patients with data on haemoglobin concentration and volume of fluids administered

The change in haemoglobin concentration from baseline was weakly associated with the volume of intravenous fluids administered in 24 h and 72 h (Fig. 2). Significant covariates included age, weight, APACHE-II score, lactate, study group and use of vasoactive agents at baseline. After adjusting for these covariates, each litre of intravenous fluid administered was associated with a statistically significant, but very small decrease in haemoglobin concentration during 24 h and 72 h (Table 3).

**Table 3:** Association between volume of intravenous fluids administered and the change in haemoglobin (Hb) concentration

Change in Hb over 24 h	Univariate analysis −1.0 (−1.4 to −0.6) Hb g/L per litre of fluid administered R^2^ = 2.5%, *P* < 0.001
	Multivariate Analysis * − 1.0 (− 1.5 to − 0.6) Hb g/L per litre of fluid administered R^2^ = 9.3%, *P* < 0.001
Change in Hb over 72 h	Univariate analysis − 1.5 (− 1.8 to − 1.2) Hb g/L per litre of fluid administered R^2^ = 8.9%, P < 0.001
	Multivariate Analysis ^#^ − 1.3 (− 1.6 to − 0.9) Hb g/L per litre of fluid administered R^2^ = 17.7%, P < 0.001

*Significant covariates were weight, APACHE-II, lactate, receiving vasoactive agents at baseline and study group. Complete data was available for 776 patients

^#^Significant covariates were age and serum lactate. Complete data was available for 594 patients

The type of fluid administered influenced the decline in haemoglobin concentration. Patients receiving only crystalloid solutions during the 72-h period (*N* = 736, 57.7%) maintained a haemoglobin concentration that was 5 (2–7) g/L higher than in those who received a combination of crystalloid and colloid fluids (*P* < 0.001). In other pre-specified sensitivity analyses, the association between haemoglobin concentration and intravenous fluid volume was not significantly altered by central venous pressure, fluid balance, serum lactate, creatinine or bilirubin.

Exploratory analysis of the change in haemoglobin at any time during the first 6 h revealed a weak association with intravenous fluid volume (*R*^2^ < 10%, *P* < 0.001) (Additional file 4: Figure S3), but not with any other resuscitation intervention analysed (Additional file 5: Figure S4).

### Association between haemoglobin and patient outcomes

Haemoglobin concentration at baseline had no relationship with mortality, even after adjusting for age, gender, APACHE-II score and serum lactate. However, a decrease in haemoglobin concentration from baseline was associated with worse patient outcomes. For each 10 g/L decrease in the haemoglobin concentration during 72 h, duration of invasive ventilation, ICU stay and hospital length of stay were longer by 14.4% (6.3–22.5%, *P* = 0.001), 16.5% (13.1–19.9%, P < 0.001) and 8.1% (5.2–10.9%, *P* < 0.001) respectively. These associations remained significant when adjusted for age, gender, APACHE-II score, Charlson Comorbidity Score, source of sepsis, lactate and vasopressor use at baseline. For each 10 g/L decrease in the haemoglobin concentration, the adjusted odds ratio of death was 1.25 (1.09–1.43, *P* = 0.001) at day 28, and 1.19 (1.05–1.39, *P* = 0.005) at day 90.

## Discussion

### Key findings

In this large cohort of patients with septic shock, haemoglobin concentration fell during the first 6-h period of resuscitation and remained low during a period of 72 h. This decline was significantly but weakly associated with the volume of intravenous fluid administered, with such volume accounting for less than 20% of the observed change in haemoglobin concentration, and a slightly greater dilutional effect with colloids. Moreover, we observed significant but very weak independent association between the baseline haemoglobin concentration and the volume of fluids subsequently administered. Finally, we observed that the decline in haemoglobin during the first 24 h and 72 h was independently associated with increased duration of ventilation, length of ICU and hospital stay and mortality.

### Comparison with previous studies

The observed decrease in haemoglobin concentration and the magnitude of change during resuscitation are in keeping with, and markedly expand the findings of other smaller studies. A single-centre study of 85 patients with septic shock, reported that haemoglobin concentration decreased by a mean of 16 g/L between samples taken in the emergency department and the first hour of ICU admission [18], and only a weak association with the amount of intravenous fluids administered. In another single-centre study of 91 heterogeneous ICU patients without evident blood loss, haemoglobin concentration decreased, mostly during the early phase of ICU admission, and was not associated with fluid balance [19].

### Possible pathophysiology

The prompt decline in haemoglobin concentration during resuscitation and the weak association with intravenous fluids implies that other factors are involved. No iatrogenic factors were identified in our study. Repeated blood sampling may have contributed to haemoglobin loss [19, 23]; however, most blood samples had already been taken at study baseline, and the decrease in haemoglobin concentration was evident within the first hour of resuscitation. A blunted erythropoietin response to anaemia, eryptosis (premature death of red blood cells), neocytolysis (removal of newly formed erythrocytes) [24], injury to red cell membranes and haemolysis can occur in sepsis [25]. These may have contributed to the decline in haemoglobin. In vitro studies have also illustrated that noradrenaline can directly inhibit erythropoiesis [26]. However, these processes would seem unlikely to explain the rapid early decrease in haemoglobin concentration noted. Furthermore, serum bilirubin concentration as a marker of haemolysis was unchanged over time and had no relationship with the change in haemoglobin. Alternative mechanisms may involve changes to the endothelial glycocalyx during sepsis [27] and interstitial fluid movement into the circulation to increase vascular volume [28]. Finally, neuroendocrine responses may also lead to haemodilution as they favour sodium and water retention at times of inadequate circulation.

The type of intravenous fluid used influenced the change in haemoglobin concentration. Patients given only crystalloid fluid maintained slightly higher haemoglobin compared with those given a mixture of crystalloid and colloid fluids. While this effect was very small, and only noted after 72 h, this observation would support the premise that a greater proportion of colloid solutions remain intravascular [29].

Unlike the increased haemoglobin concentrations noted in many experimental models of sepsis, we did not observe markedly high concentrations in this cohort of patients. Although in this study we did not control for timing and severity of disease, it suggests there may be species-specific differences in the response to sepsis. Experimental sepsis models may have greater loss of plasma fluid or liberation of red blood cells from reticuloendothelial organs such as the spleen, liver and bone marrow.

### Clinical implications

Our findings imply that an early decrease in haemoglobin concentration in patients with septic shock is ubiquitous and largely due to factors unrelated to the administered volume of intravenous fluid. They also imply that the decrease in haemoglobin concentration is independently associated with longer length of stay (ICU and hospital) and greater mortality (day 28 and day 90) with the adjusted odds ratio for 28-day mortality increased by 25% for only a modest (10 g/L) decrease in haemoglobin. Accordingly, the early decline in haemoglobin concentration in septic shock appears to be a clinically important marker of illness severity.

### Strengths and limitations

This is the largest and most detailed study to have specifically assessed the change in haemoglobin concentration in patients resuscitated from septic shock. The study cohort was recruited from multiple hospitals, relatively few patients were excluded for having received red blood cells and no patients were lost to follow up. Clinically relevant confounders, including ARISE study group allocation, were considered in multivariate analyses. Estimates of association were relatively precise, particularly given the large study cohort. The findings are generalisable to other patients with septic shock who are not bleeding, but not necessarily to patients with other critical illnesses.

A number of study limitations need to be considered. This post-hoc analysis was not defined in the study design for ARISE. This exploratory analysis contains multiple comparisons and is at risk of identifying random associations. Nevertheless we did not identify any association that would be considered spurious, and the low *P* values make a type 1 error unlikely. Measures of red blood cell concentration (such as haematocrit) may better reflect haemoconcentration or haemodilution than haemoglobin. While haematocrit was assessed in ARISE, most study sites did not measure it. Thus, haemoglobin concentration was used in this study. Other markers of haemolysis (e.g. lactate dehydrogenase) or the marrow response to anaemia (e.g. reticulocytes) were not available. Volume kinetic studies may have provided insights into the distribution of administered fluids, but this type of analysis was not possible with the data available. Finally, incomplete data on urine volume precluded reliable estimates of fluid balance and changes in patient weight were not available for the cohort. However, sensitivity analysis using the available fluid balance data did not alter the interpretation of the study.

## Conclusions

Haemoglobin concentration decreases in patients resuscitated from septic shock. This is apparent early in resuscitation and persists over the following 72 h, but is only weakly associated with the volume of intravenous fluid administered. Other disease processes are likely to account for most of the change in haemoglobin concentration in septic shock, a phenomenon independently associated with increased length of hospital stay and mortality.

## Additional files

Additional file 1: **Figure S1.** Hemodynamic parameters and urine output at each time point in the cohort study of patients enrolled in the ARISE trial. (PDF 40 kb)

Additional file 2: **Figure S2.** Haemoglobin concentration at 0, 24 and 72 h separated by ARISE study groups (usual care vs. EGDT). (PDF 38 kb)

Additional file 3: **Table S1.** Association between haemoglobin (Hb) concentration at enrolment into ARISE and subsequent volume of intravenous fluids administered. (PDF 452 kb)

Additional file 4: **Figure S3.** Exploratory analysis of the change in haemoglobin according to the volume of intravenous fluid administered during the first 6 h of resuscitation. (PDF 128 kb)

Additional file 5: **Figure S4.** Exploratory analysis of the change in haemoglobin according to resuscitation intervention applied at each hour (insertion of intra-arterial line, central venous catheter, mechanical ventilation, use of a vasoactive infusion). (PDF 66 kb)

## Abbreviations

ANZICS: Australian and New Zealand Intensive Care Society
APACHE-II: Acute Physiology and Chronic Health Evaluation II
ARISE: Australasian Resuscitation in Sepsis Evaluation
EGDT: Early goal-directed therapy
Hb: Haemoglobin
ICU: Intensive Care Unit
IQR: Interquartile range
