# Supplementary material for: Haemoglobin concentration and volume of intravenous fluids in septic shock in the ARISE trial
Source: Crit Care. 2018 May 3;22:118. doi: 10.1186/s13054-018-2029-6 (PMC5934793; doi:10.1186/s13054-018-2029-6)

Figure S1: Haemodynamic parameters and urine output at each time point in the cohort study of patients enrolled in the ARISE trial. Data is presented as median and inter-quartile range. N=number of patients with data at each time point.

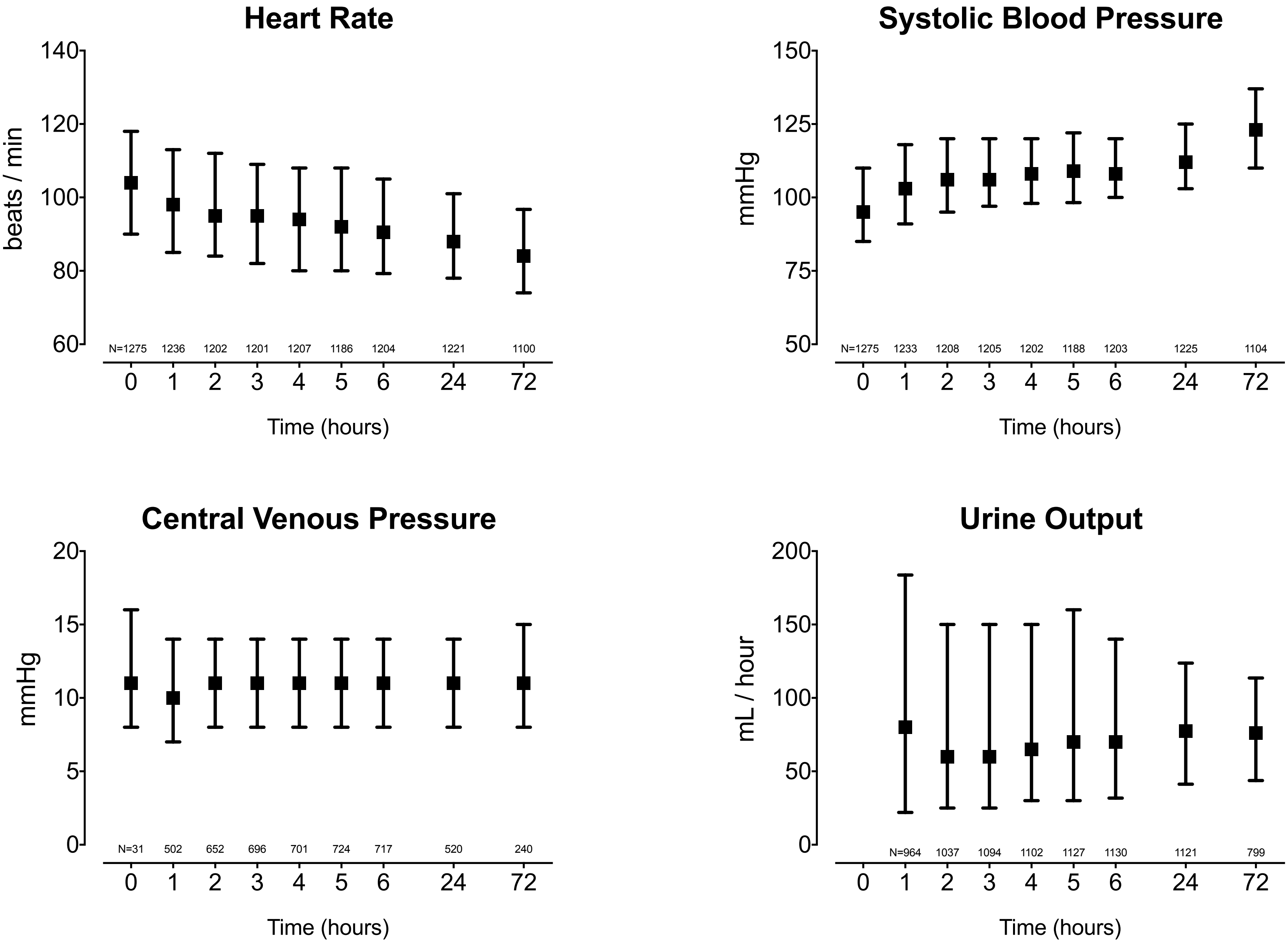

Supplement: Supplementary file 1 — Figure S1. Hemodynamic parameters and urine output at each time point in the cohort study of patients enrolled in the ARISE trial. (PDF 40 kb) [file 13054_2018_2029_MOESM1_ESM.pdf]
