# Supplementary material for: Haemoglobin concentration and volume of intravenous fluids in septic shock in the ARISE trial
Source: Crit Care. 2018 May 3;22:118. doi: 10.1186/s13054-018-2029-6 (PMC5934793; doi:10.1186/s13054-018-2029-6)

Figure S2: Haemoglobin concentrations for the cohort at each time point according to ARISE study group.   Data is presnted as median, boxes as interquartile range and whiskers as range.

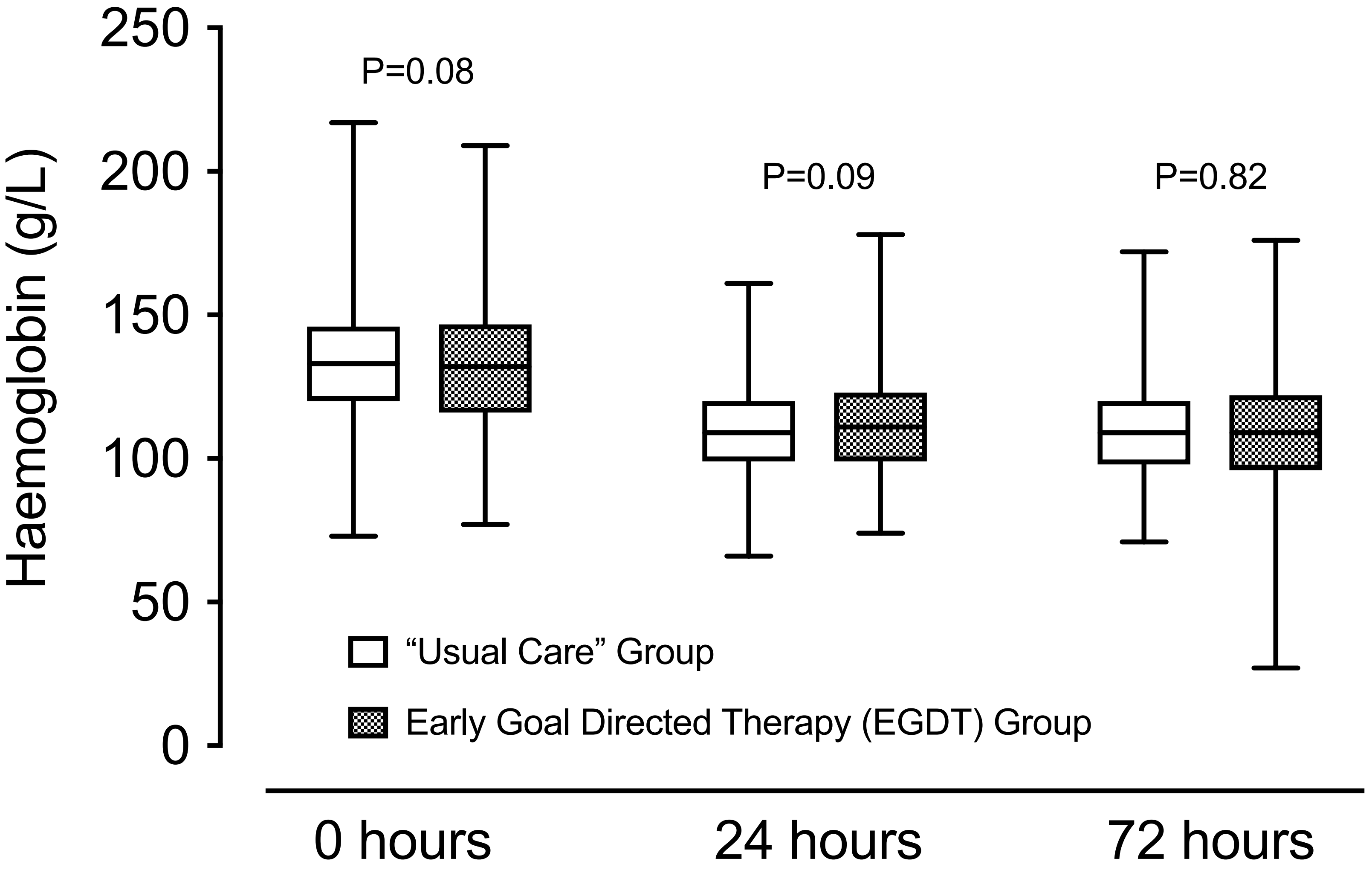

Supplement: Supplementary file 2 — Figure S2. Haemoglobin concentration at 0, 24 and 72 h separated by ARISE study groups (usual care vs. EGDT). (PDF 38 kb) [file 13054_2018_2029_MOESM2_ESM.pdf]
