# Supplementary material for: Haemoglobin concentration and volume of intravenous fluids in septic shock in the ARISE trial
Source: Crit Care. 2018 May 3;22:118. doi: 10.1186/s13054-018-2029-6 (PMC5934793; doi:10.1186/s13054-018-2029-6)
Supplement: Supplementary file 3 — Table S1. Association between haemoglobin (Hb) concentration at enrolment into ARISE and subsequent volume of intravenous fluids administered. (PDF 452 kb) [file 13054_2018_2029_MOESM3_ESM.pdf]

Table S1: Association between haemoglobin (Hb) concentration at enrolment into ARISE and subsequent volume of intravenous fluids administered.

**Fluid volume administered over 6 hours**	Univariate analysis<br>5 mL more fluid administered per gram Hb / L<br>R^2 <1%, P=0.007<br><br>Multivariate Analysis *<br>6 mL more fluid administered per gram Hb / L<br>R^2 = 4.2%, P<0.001
**Fluid volume administered over 24 hours**	Univariate analysis<br>12 mL more fluid administered per gram Hb / L<br>R^2 = 1.3 %, P<0.001<br><br>Multivariate Analysis #<br>13 mL more fluid administered per gram Hb / L<br>R^2 = 3.8%, P<0.001
**Fluid volume administered over 72 hours**	Univariate analysis<br>18 mL more fluid administered per gram Hb / L<br>R^2 = 1.1 %, P<0.001<br><br>Multivariate Analysis #<br>22 mL more fluid administered per gram Hb / L<br>R^2 = 4.4%, P<0.001

* Significant covariates were age, APACHE-II and study group. Complete data was available for 1,205 patients.

# Significant covariates were age and APACHE-II. Complete data was available for 1,245 patients.
