# Supplementary material for: Haemoglobin concentration and volume of intravenous fluids in septic shock in the ARISE trial
Source: Crit Care. 2018 May 3;22:118. doi: 10.1186/s13054-018-2029-6 (PMC5934793; doi:10.1186/s13054-018-2029-6)

Figure S3: Change in haemoglobin concentration vs. volume of intravenous fluid at each hour during the first six hours of resuscitation in a cohort of patients enrolled in the ARISE trial. Data is presented as scatter plots with regression line.

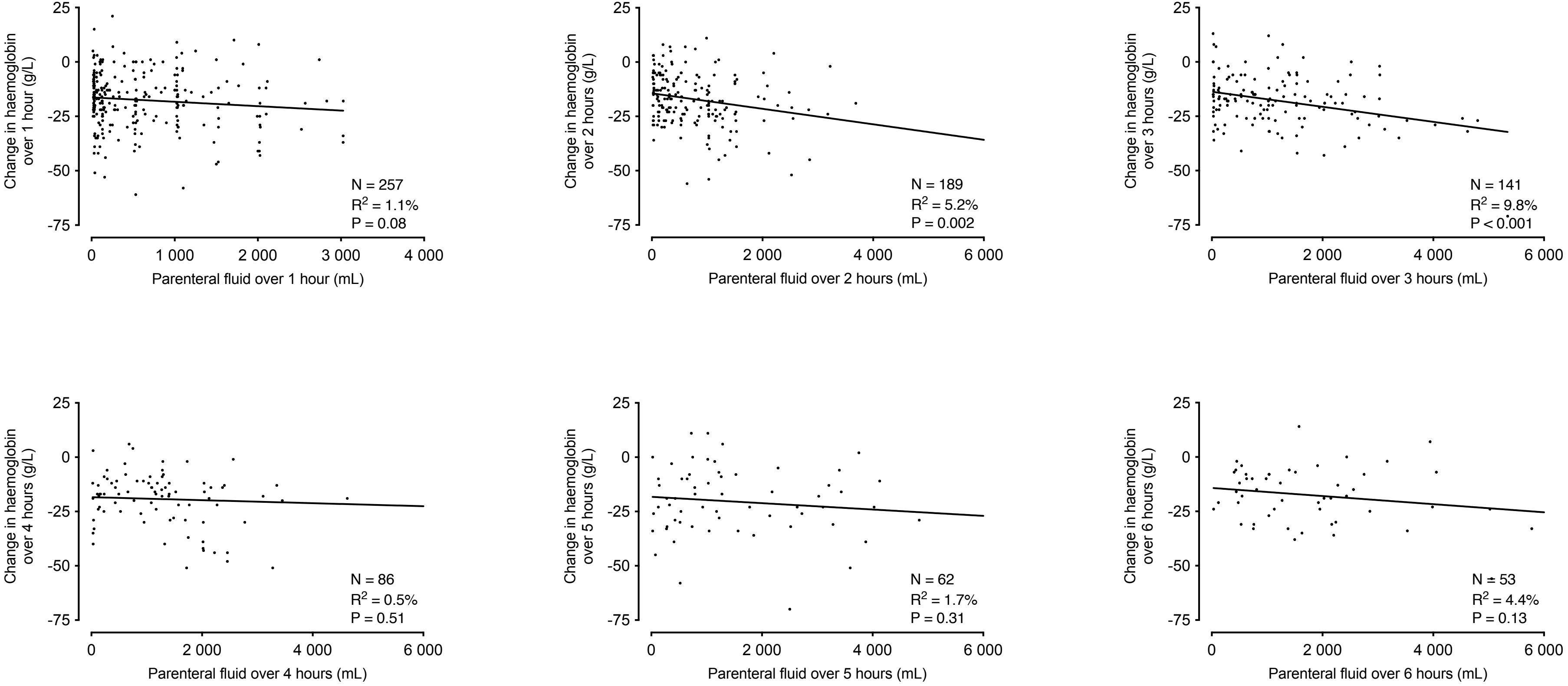

Supplement: Supplementary file 4 — Figure S3. Exploratory analysis of the change in haemoglobin according to the volume of intravenous fluid administered during the first 6 h of resuscitation. (PDF 128 kb) [file 13054_2018_2029_MOESM4_ESM.pdf]
