# Supplementary material for: Haemoglobin concentration and volume of intravenous fluids in septic shock in the ARISE trial
Source: Crit Care. 2018 May 3;22:118. doi: 10.1186/s13054-018-2029-6 (PMC5934793; doi:10.1186/s13054-018-2029-6)

Figure S4: Change in haemoglobin concentation for the cohort of patients enrolled in the ARISE trial separted accoriding to timing of resuscitation intervention during the first six hours.

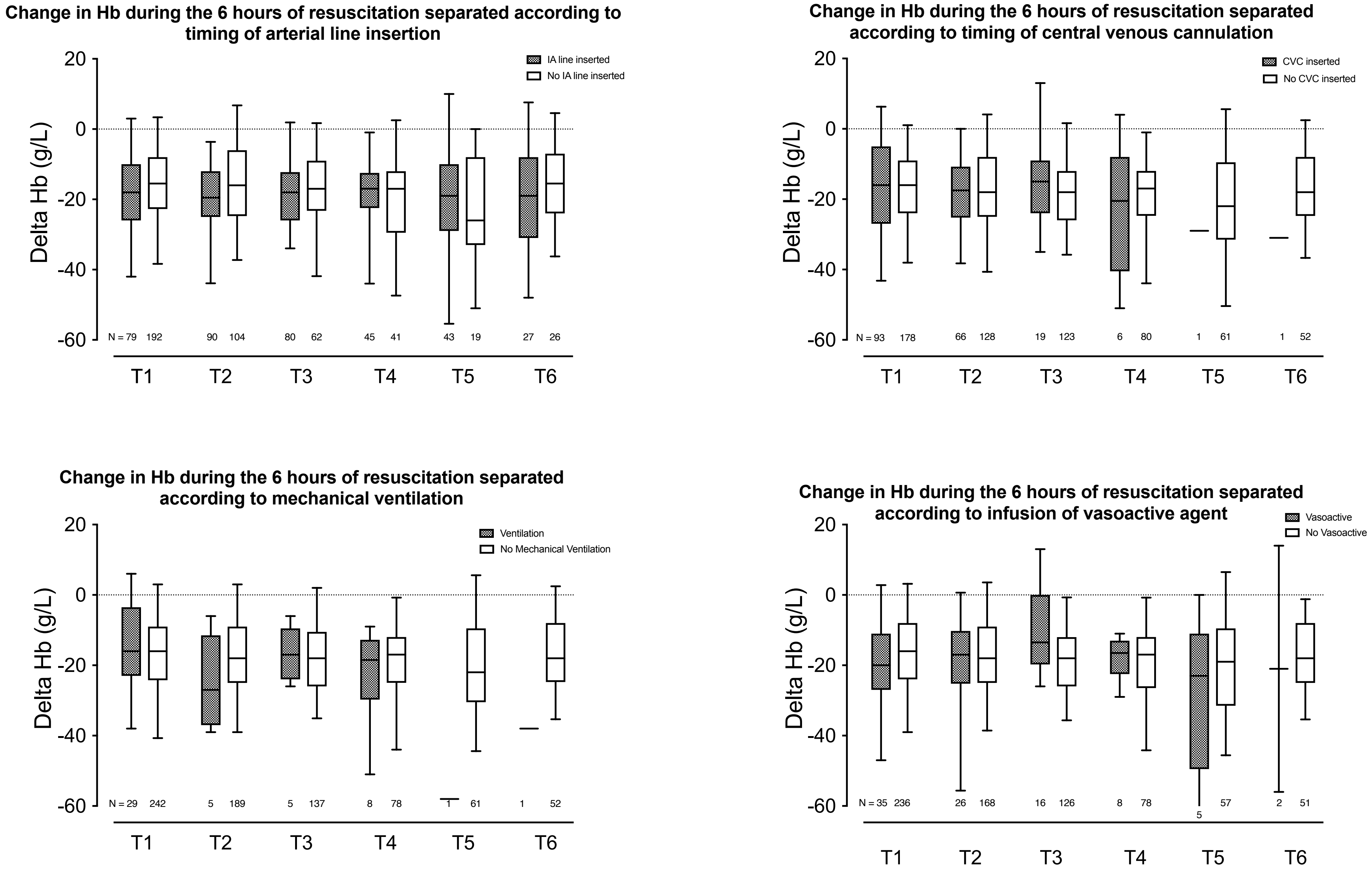

Supplement: Supplementary file 5 — Figure S4. Exploratory analysis of the change in haemoglobin according to resuscitation intervention applied at each hour (insertion of intra-arterial line, central venous catheter, mechanical ventilation, use of a vasoactive infusion). (PDF 66 kb) [file 13054_2018_2029_MOESM5_ESM.pdf]
